# Circulating cell-free DNA methylation analysis of pancreatic cancer patients for early noninvasive diagnosis

**DOI:** 10.3389/fonc.2025.1552426

**Published:** 2025-03-10

**Authors:** Wenzhe Hu, Xudong Zhao, Nan Luo, Mengmeng Xiao, Feng Feng, Yuan An, Jianfei Chen, Long Rong, Yinmo Yang, Jirun Peng

**Affiliations:** ^1^ Department of Surgery, Beijing Shijitan Hospital, Capital Medical University, Beijing, China; ^2^ School of Oncology, Capital Medical University, Beijing, China; ^3^ Department of Endoscopy Center, Peking University First Hospital, Peking University, Beijing, China; ^4^ Ninth School of Clinical Medicine, Peking University, Beijing, China; ^5^ Department of General Surgery, Peking University People’s Hospital, Peking University, Beijing, China; ^6^ Department of General Surgery, Peking University First Hospital, Peking University, Beijing, China

**Keywords:** circulating cell-free DNA, DNA methylation, pancreatic cancer, early noninvasive diagnosis, hepatocellular carcinoma, colorectal cancer, gastric cancer

## Abstract

**Background:**

Aberrant hypermethylation of genomic DNA CpG islands (CGIs) is frequently observed in human pancreatic cancer (PAC). A plasma cell-free DNA (cfDNA) methylation analysis method can be utilized for the early and noninvasive detection of PAC. This study also aimed to differentiate PAC from other cancer types.

**Methods:**

We employed the methylated CpG tandem amplification and sequencing (MCTA-Seq) method, which targets approximately one-third of CGIs, on plasma samples from PAC patients (n = 50) and healthy controls (n = 52), as well as from cancerous and adjacent noncancerous tissue samples (n = 66). The method’s efficacy in detecting PAC and distinguishing it from hepatocellular carcinoma (HCC), colorectal cancer (CRC), and gastric cancer (GC) was evaluated. Additionally, a methylation score and typing system for PAC was also established.

**Results:**

We identified a total of 120 cfDNA methylation biomarkers, including *IRX4*, *KCNS2*, and *RIMS4*, for the detection of PAC in blood. A panel comprising these biomarkers achieved a sensitivity of 97% and 86% for patients in the discovery and validation cohorts, respectively, with a specificity of 100% in both cohorts. The methylation scoring and typing systems were clinically applicable. Furthermore, we identified hundreds of differentially methylated cfDNA biomarkers between PAC and HCC, CRC, and GC. Certain combinations of these markers can be used in a highly specific (approximately 100%) algorithm to differentiate PAC from HCC, CRC, and GC in blood.

**Conclusions:**

Our study identified cfDNA methylation markers for PAC, offering a novel approach for the early, noninvasive diagnosis of PAC.

## 1 Introduction

In the realm of cancer research, alterations in DNA methylation have emerged as highly promising targets for the development of potent diagnostic biomarkers (1–4). The analysis technologies for DNA methylation can be generally classified into two main categories: typing and profiling. Typing focuses on identifying specific methylation patterns at particular loci, while profiling aims to comprehensively map the methylation status across the entire genome. To obtain more comprehensive and accurate genomic insights, the majority of researchers, including our team, place a high priority on profiling techniques (5–11). Globally, pancreatic cancer (PAC) stands as the seventh leading cause of cancer - related mortality (12). The early symptoms of PAC are often rather subtle and nonspecific. They typically manifest as mild abdominal pain, which can easily be mistaken for common digestive issues, or as relatively minor problems with blood glucose control. As a result, the majority of patients are unfortunately diagnosed at an advanced stage, resulting in a 5-year survival rate of only 12% (13). Currently, the most commonly clinically available biomarker for PAC is the serum level of carbohydrate antigen 19-9 (CA19-9), which lacks both diagnostic power and specificity in the early stage of the disease (14, 15). Considering the relatively low incidence of PAC in the general population, the risk of overdiagnosis during screening efforts must be carefully considered (16). Overdiagnosis can lead to unnecessary medical interventions, causing physical and psychological harm to patients, as well as wasting valuable medical resources (17–19). So, there is an urgent need to identify new biomarkers or a panel of biomarkers to address the gaps in the early diagnosis and treatment of PAC. Although previous studies have explored the potential of circulating cell - free DNA (cfDNA) in differentiating various cancer diseases, the research specifically focused on PAC remains relatively limited (20–22). This limited research on cfDNA in the context of PAC creates an opportunity for further investigation to potentially uncover novel diagnostic and prognostic markers for this deadly disease.

We previously developed methylated CpG tandem amplification and sequencing (MCTA-Seq), a high-throughput platform capable of investigating thousands of CpG islands (CGIs) in a single blood-based experiment (23). In this study, we applied MCTA-Seq to analyses 168 clinical samples, comprising 66 tissue samples and 102 plasma samples from patients with PAC and healthy controls. Our findings have the potential to advance the development of cfDNA methylation biomarkers and enhance the clinical detection of PAC.

## 2 Materials and methods

### 2.1 Study design and patient cohorts

Patients and healthy controls were carefully recruited from the Department of Surgery at Beijing Shijitan Hospital, Capital Medical University, China. All patients had a definite postoperative pathological diagnosis, and all normal healthy controls were normal people who had no history of malignant tumors. The study protocol was approved by the Ethics Committee of Beijing Shijitan Hospital (No. IIT2024-133-002). The Ethics Committee’s review was comprehensive, covering all aspects of the study, from the recruitment of participants to the handling and storage of samples, and the subsequent data analysis. This approval was essential to ensure that the study was conducted in an ethical manner, protecting the rights and well - being of all participants. Prior to their inclusion in the study, all participants were fully informed about the nature, purpose, and potential risks and benefits of the study. They were provided with detailed written materials explaining the procedures involved in the collection of samples, which included blood draws, tissue biopsies (if applicable), and how these samples would be used in subsequent analyses. Ultimately, we collected a total of 51 plasma samples and 66 tissue samples from patients, along with 52 plasma samples from healthy controls. Rigorous quality control measures were implemented to ensure the reliability of our data (24–26). Specifically, any sample with a total molecular count of less than 10,000 was excluded from further analysis. In line with these criteria, only one patient’s plasma sample failed to meet the threshold and was thus excluded from the study.

Subsequently, the remaining 102 plasma samples were randomly partitioned into two groups according to a 6:4 ratio. Cohort I, which consisted of 30 patient cases and 31 healthy controls, was designated as the discovery set. This set was primarily used to explore and identify potential biomarkers or patterns related to the research objective. On the other hand, Cohort II, comprising 20 patient cases and 21 healthy controls, served as the validation set. Its purpose was to independently verify the findings obtained from the discovery set, thereby enhancing the robustness and generalizability of our research results.

### 2.2 Sample DNA MCTA-Seq library preparation, and data processing

The tissue samples, each weighing approximately 20 milligrams, were immediately cryopreserved at -80°C following a thorough rinse with isotonic saline. Concurrently, 5 to 10 milliliters of blood were collected and subjected to centrifugation at 4°C within a 6-hour window. DNA extraction and MCTA-Seq library preparation were subsequently performed according to previously described methods (23). The prepared libraries were sequenced via the Illumina HiSeq platform by Novogene Corporation, with a sequencing depth of 0.5 gigabytes per nanogram of circulating cell-free DNA for plasma samples or 2 gigabytes for each tissue sample, yielding 150-base pair-end reads. The sequencing data were processed as previously described (27, 28).

### 2.3 Discovery of plasma methylation biomarkers via genome-wide expression profiling

Building upon the methodologies of previous studies, we established criteria for the selection of tumor-specific methylation biomarkers for the diagnosis of PAC. These criteria include (1) significant differential hypermethylation in PAC tissues compared with noncancerous tissues (*P* < 0.01); (2) proximity to CGCGCGG motifs, with the biomarkers located less than 60 base pairs away; (3) a high methylation level of at least 10 methylated alleles per million mapped reads (MEPM); and (4) a low background methylation frequency in normal plasma, which should be less than 5% to ensure specificity. To validate these markers, we employed Monte Carlo cross-validation, which involved randomly dividing the cohorts into training and test sets 1000 times, and then quantifying the number of mCGCGCGG-CpG sites exhibiting positive methylation values.

For further refinement of these biomarkers to differentiate PAC from other malignancies, such as hepatocellular carcinoma (HCC), colorectal cancer (CRC), and gastric cancer (GC), we refined our selection criteria: (1) significant differential methylation between the two types of tumor tissues (*P* < 0.01, fold change >2); (2) a minimum of 5 MEPM values in tumor tissues; and (3) a consistently low methylation frequency in normal plasma across the different cancer types, with the threshold set at less than 5%.

### 2.4 Construction of the methylation scoring and typing system

Consensus clustering is a method for class discovery that aims to identify unknown clusters composed of items sharing similar intrinsic features, as described in study (29–35). In our research, all PAC tissue samples were subjected to clustering analysis. The optimal number of clusters was determined by examining the cumulative distribution function (CDF). Similarly, plasma samples were also analyses via an unsupervised clustering algorithm. To pinpoint the most significant features among plasma methylated biomarkers and mitigate the risk of overfitting, we employed least absolute shrinkage and selection operator (LASSO) regression (36, 37). All these identified features were subsequently incorporated into a logistic regression analysis. The methylation score was calculated via the following formula:


$$Methylation\;\text{score}=\;\sum^{\;}\left(\text{Coefi}\times \text{Ai}\right)$$

In this formula, Coefi denotes the regression coefficient of the logistic model for each biomarker, i represents the individual biomarker, and Ai signifies the MEPM value.

### 2.5 Establishment of a tumor type classifier

In alignment with previous studies, for each plasma sample under evaluation, we conducted an assessment of both PAC-specific and non-PAC diagnostic panels. The diagnostic process proceeded as follows: samples were classified as non-cancerous only when both the PAC panel and the non-PAC panel yielded negative results. A positive result from either panel led to the classification of the sample as cancerous, with the specific cancer type determined by the panel that tested positive. The non-PAC panel specifically identifies HCC, CRC, and GC, with diagnostic criteria established in prior research (28). In instances where multiple panels returned positive results, a secondary evaluation was performed, prioritizing the panel with the highest percentage of positively methylated CpG sites to determine the final diagnosis. If the above process results in an indeterminate outcome—where two or more panels exhibit an equal proportion of positively methylated CpG sites—a definitive diagnosis cannot be established. While this scenario is theoretically possible, it is exceptionally rare in practice. The selection of biomarkers and the establishment of their cut-off values were guided by tissue analysis results and validated using an independent cohort of normal plasma samples. This methodology was implemented to reduce the risk of bias and enhance the reliability of the diagnostic process.

### 2.6 Statistical analysis

For our analysis, we utilized custom R scripts and R packages to construct a variety of visualizations and statistical measures. These include principal component analysis (PCA) for dimensionality reduction, heatmaps for data representation, box plots for distribution analysis, and scatter plots for exploring relationships between variables. Additionally, we employed circle plots and uniform manifold approximation and projection (UMAP) for advanced visualization techniques. To quantify the performance of our diagnostic models, we calculated the area under the curve (AUC). For unsupervised clustering, we applied algorithms to identify natural groupings within the data. P < 0.05 was set as the benchmark for determining statistical significance in our study (38–41).

## 3 Results

### 3.1 Identification of possible tissue and plasma candidates

**Figure 1:**
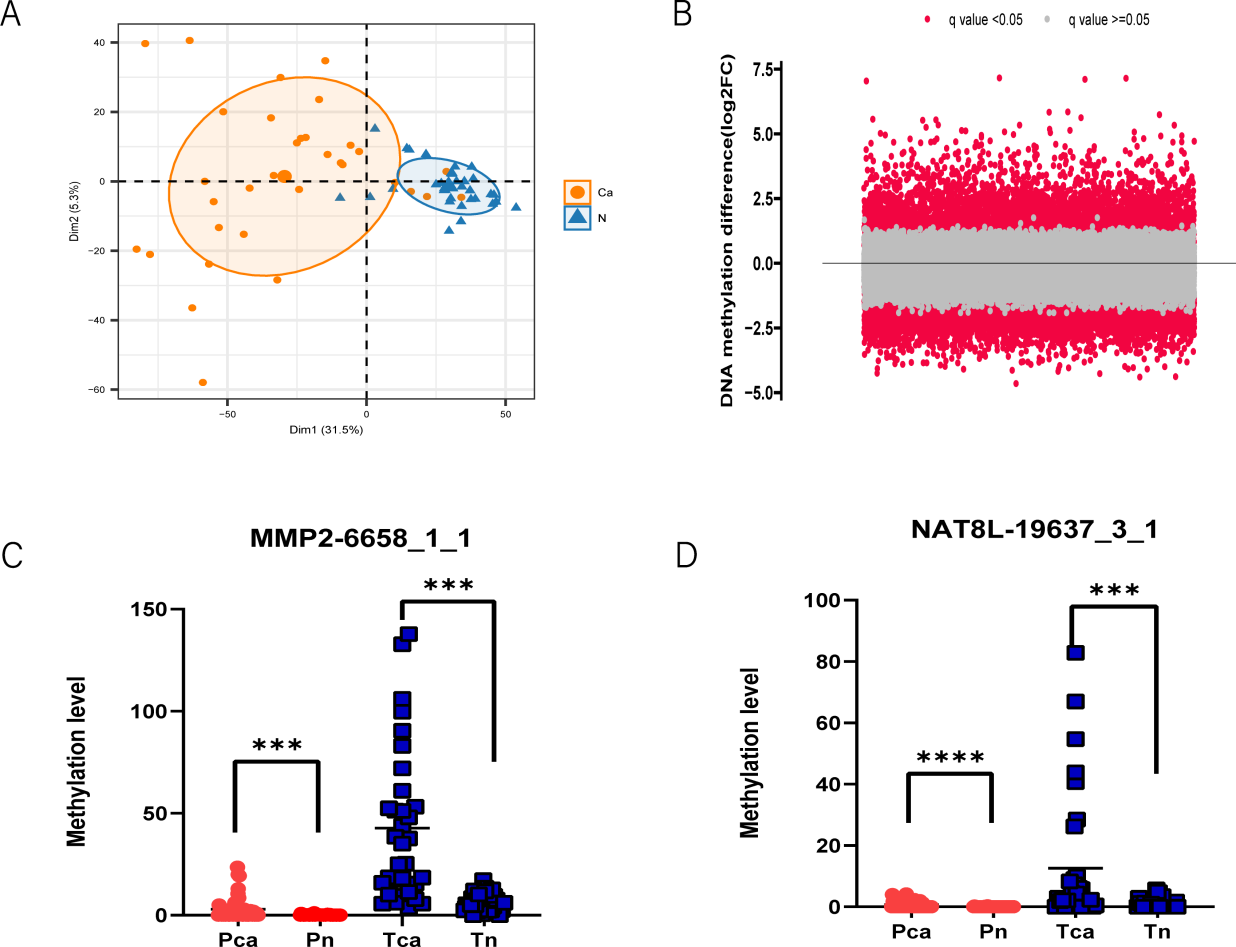
MCTA-Seq screened methylated cfDNA biomarkers for PAC. **(A)** Principal component analysis revealed that PAC cancer tissues have different DNA methylation patterns than adjacent normal tissues. **(B)** Volcano plot showing differentially methylated CpG sites in the plasma of PAC patients and the control group. The q value indicates the false discovery rate. *MMP2*-6658_1_1 **(C)** and *NAT8L*-19637_3_1 **(D)** are representative CpG site biomarkers. ***P < 0.001; ****P < 0.0001; two-tailed MWW test.

We applied MCTA-Seq to detect methylation in the tissues and plasma of PAC patients to screen for cancer-specific diagnostic biomarkers. For the tissue samples, we included 33 pairs of PAC tissues and matched adjacent noncancerous tissues. A difference analysis between cancerous and noncancerous tissues identified 4615 differentially methylated CpG sites in cancer (two-tailed Mann-Whitney U-test, false discovery rate < 0.05, mean methylation fold change > 2). PCA also revealed that most (31 of 33) of the PAC cancerous tissues were distinguished from the noncancerous tissues (**Figure 1A**). These results indicate that methylation changes significantly during the carcinogenesis of PAC, which is consistent with our previous studies in other tumors (27, 28).

Expanding our investigation to plasma samples, we examined a total of 30 patient plasma samples and 31 normal control plasma samples in the discovery cohort. A comparison between the plasma of patients and controls revealed 1323 upregulated and 2619 downregulated methylated CpG sites (false discovery rate < 0.05, mean methylation fold change >2, two-tailed Mann–Whitney U-test) (**Figure 1B**). In general, the mCGCGCGG-CpGs that are differentially methylated in both tissues and plasma are most likely to be included in the final diagnostic panel. For mCGCGCGG-CpGs that are differentially methylated only in tissues or plasma, the reason may be their different genomic locations. The demographic data of the above participants are presented in **Supplementary Table S1**, of the online Data Supplement. All MCTA-Seq data analyses were based on the fully methylated molecule (FMM) algorithm (27). Among the biomarkers identified, several have been recognized as potential diagnostic indicators for PAC, including *IRX4, PRKCB*, and *CLEC11A*. A significant portion of these biomarkers, however, were newly identified in our study, such as *MMP2, NAT8L*, and *GFRA4* (**Figures 1C, D**). These novel markers demonstrate a superior signal-to-raise ratio compared with previously known markers, suggesting their enhanced potential for accurate PAC diagnosis.

### 3.2 Establishment and evaluation of the PAC classifier

In clinical practice, the ability to differentiate between benign and malignant lesions at the earliest possible stage is critically important, as it directly influences the choice of treatment strategy. Given the significance of this issue, there is an urgent need to develop a reliable classifier capable of accurately identifying patients with PAC. Leveraging PAC tissues and normal plasma samples from our previous study (GSE124600) as an independent dataset, we employed a method called additive positivity to select candidate biomarkers. This approach involved counting the number of mCGCGCGG-CpGs with positive methylation values.

**Figure 2:**
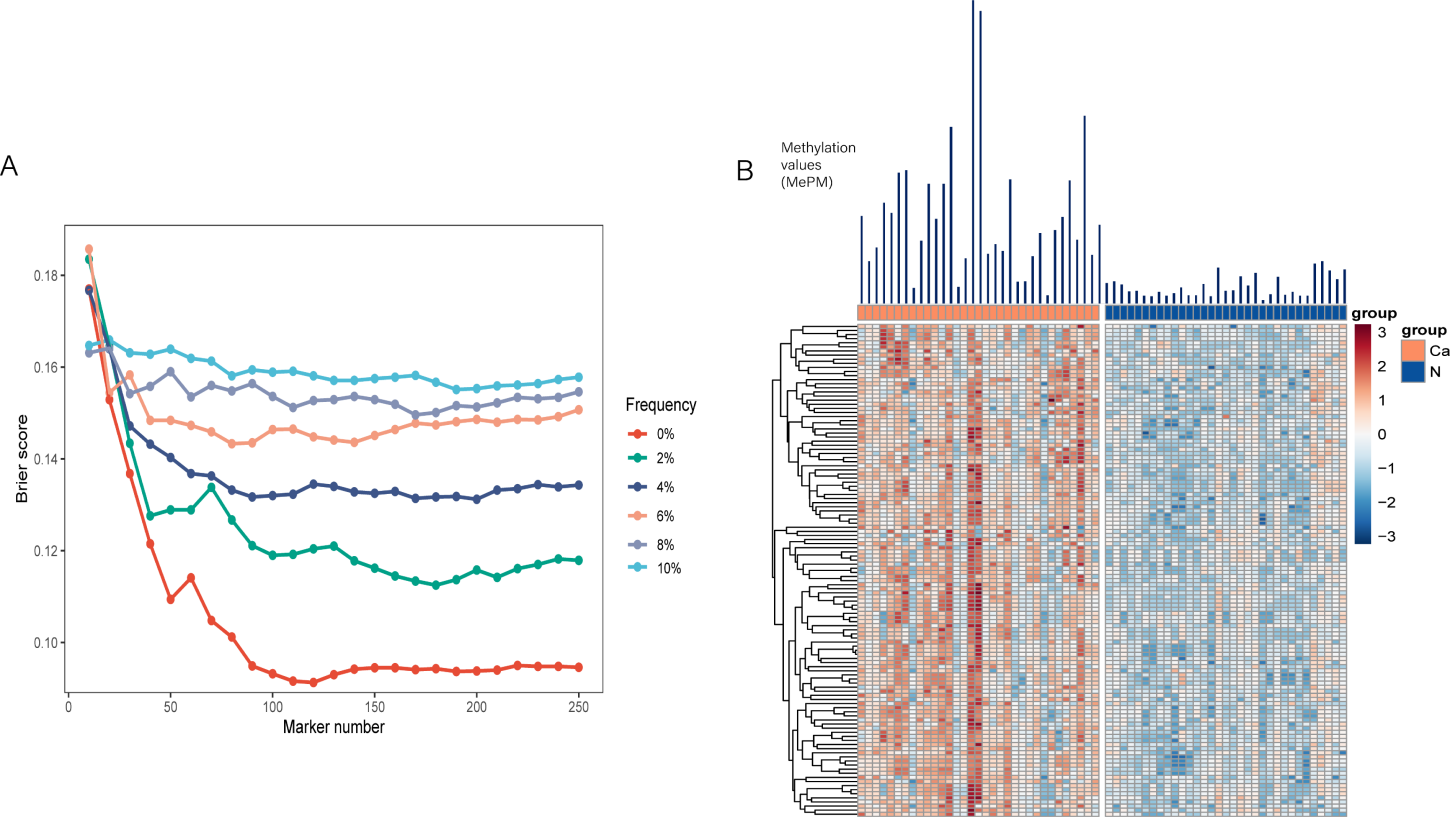
Establishment of the PAC classifier. **(A)** Brier scores of the counting model trained from variables including the methylation frequency in the normal plasma of the preselection set and the total number of markers. **(B)** Heatmap of the 120 mCGCGCGG-CpGs in PAC and adjacent normal tissues.

To ensure robustness, we randomly divided the discovery cohort into training and test sets 1,000 times using Monte Carlo cross-validation. Through this process, a panel of 120 mCGCGCGG-CpGs demonstrated the best classification performance (**Figure 2A**). The Brier score, which quantifies the likelihood of prediction error, decreased progressively as more biomarkers were included in the fre0 group, reaching its lowest point between 100 and 150 markers. Detailed information about the 120 mCGCGCGG-CpGs included in the classifier is provided in **Supplementary Table S2** of the online Data Supplement. These mCGCGCGG-CpGs exhibit hypermethylation in both tissues and plasma of cancer patients compared to normal tissues and control plasma, ensuring high sensitivity in model detection (**Figure 2B**). The total methylation values of the 120 mCGCGCGG-CpGs in PAC tissues ranged from 247 to 9,232, with a median of 2,243, while in normal tissues, the range was 10 to 800, with a median of 268. Notably, the number of positively methylated mCGCGCGG-CpGs in the plasma of PAC patients ranged from 9 to 103 (median: 34), compared to 0 to 10 (median: 2) in controls. This significant difference underscores the potential of this classifier for screening high-risk populations for PAC. The development of this classifier represents a promising step toward early and accurate detection of PAC, which is crucial for improving patient outcomes through timely and appropriate treatment interventions.

**Figure 3:**
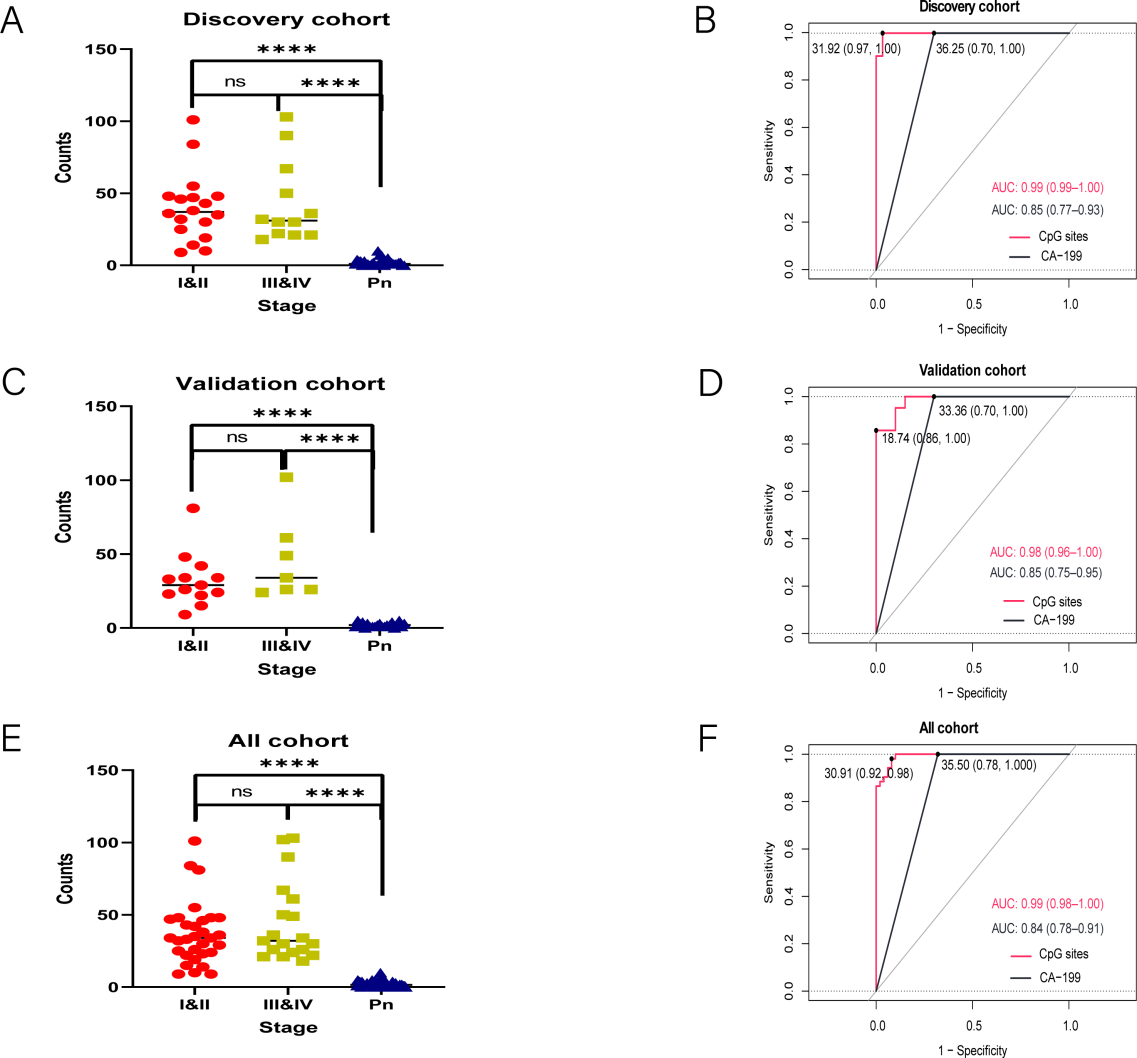
Detection of the 120 mCGCGCGG-CpGs in plasma from patients with PAC and controls. Positive counts of the 120 mCGCGCGG-CpGs in PAC patients and normal controls **(A, C, E)**. Receiver operating characteristic curves for the 120-loci cfDNA methylation panel and plasma CA19-9 alone for distinguishing PACs from healthy controls in the discovery **(B)**, and validation **(D)** cohorts and all cohorts **(F)** are shown. The P values were computed via the two-tailed Mann–Whitney U-test. ****P < 0.0001; two-tailed MWW test. ns, not significant.

To assess the diagnostic performance of the 120-mCGCGCGG-CpGs panel, we analyzed positive counts and AUC values in both the discovery and validation cohorts. In the discovery cohort (**Figure 3A**), the positive counts of the 120 mCGCGCGG-CpGs were significantly higher in patients with both early-stage (stages I&II) and advanced-stage (stages III&IV) PAC compared to controls. The median counts were 0 for controls, 37 for stages I&II, and 31 for stages III&IV (*P* < 0.0001, two-tailed Mann–Whitney U-test). Receiver operating characteristic (ROC) curve analysis showed that the 120-mCGCGCGG-CpGs panel achieved an AUC value of 0.99 (95% CI, 0.99-1.00) for all-stage samples, outperforming CA19-9, which had an AUC of 0.85 (95% CI, 0.77-0.93) (**Figure 3B**).

Similarly, in the validation cohort (**Figures 3C, D**), the positive counts were significantly higher in PAC plasma samples than in controls. The median counts were 29 for stage I&II, 34 for stage III&IV, and 0 for controls (*P* < 0.001, two-tailed Mann–Whitney U-test). The AUC values for the 120-mCGCGCGG-CpGs panel and CA19-9 were 0.98 (95% CI, 0.96-1.00) and 0.87 (95% CI, 0.78-0.97), respectively, further confirming the panel’s superior diagnostic performance. When combining the training and validation cohorts, the 120-mCGCGCGG-CpGs panel maintained an exceptional AUC value of 0.99 (95% CI, 0.99-1.00) for diagnosing PAC, while CA19-9 had an AUC of 0.85 (95% CI, 0.77-0.93) (**Figures 3E, F**). These results highlight the robustness and high diagnostic accuracy of the 120-mCGCGCGG-CpGs panel across different cohorts and stages of PAC, underscoring its potential as a reliable tool for early and accurate detection of pancreatic adenocarcinoma.

### 3.3 Methylation scoring and typing system

**Figure 4:**
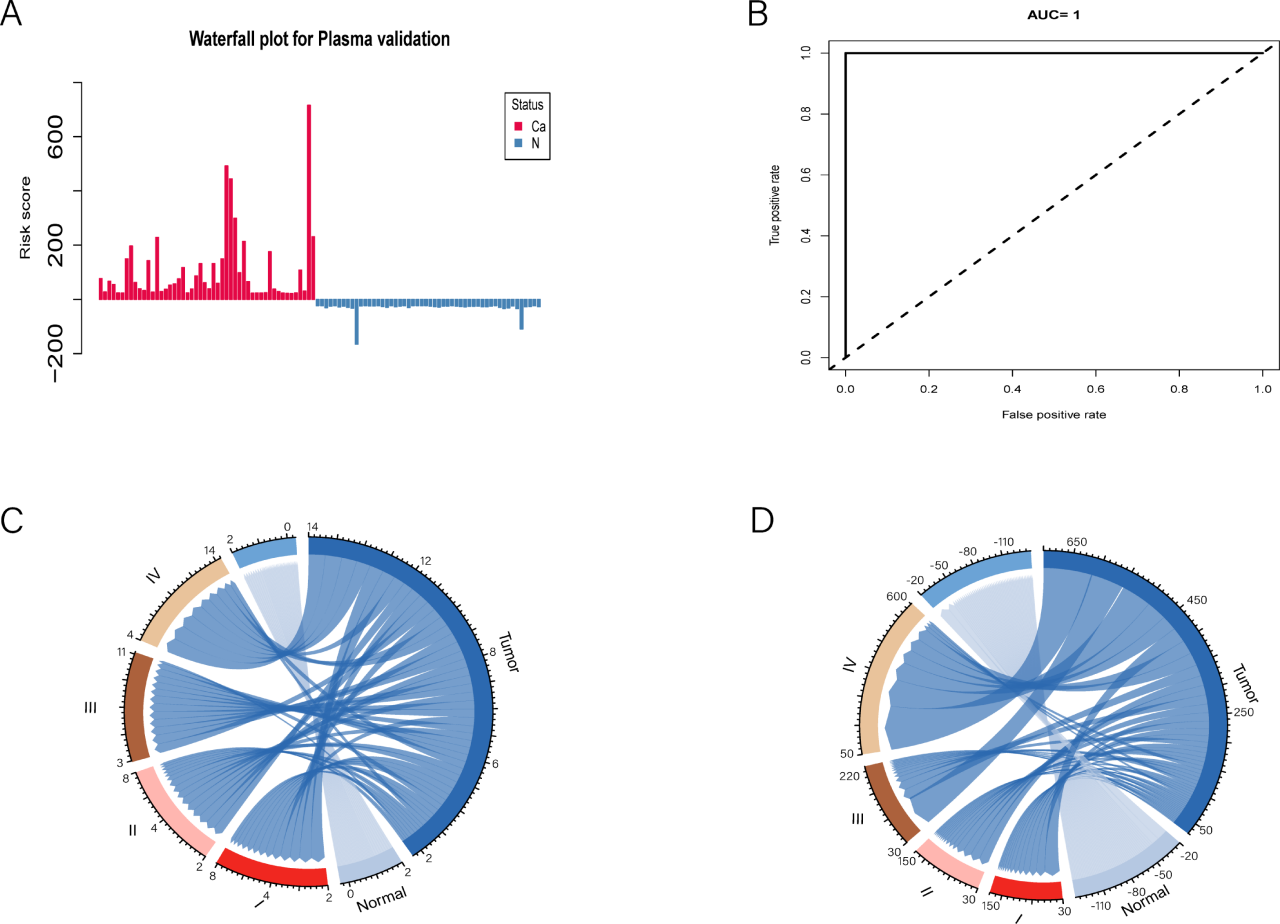
Simplifying to 14 mCGCGCGCGG-CpGs for risk assessment and disease staging in PAC patients. **(A)** The waterfall plot illustrates the risk probability distribution between PAC and normal plasmas. **(B)** ROC curve analysis revealed the performance of the selected 14-cfDNA panel in all PACs and normal plasmas. **(C, D)** The positive numbers and total methylation values of these 14 mCGCGCGCGG-CpGs in different tumor stages were significantly different.

Following the risk score calculation, a waterfall plot analysis was conducted to categorize the cases on the basis of their scores (**Figure 4A**). Our model demonstrated exceptional discrimination between patients and controls, correctly identifying all 52 patients (100.0%) as true positives and all 52 controls (100.0%) as true negatives (**Figure 4B**). Notably, the number of positive CpG sites and their total methylation values varied significantly across different tumor stages (**Figures 4C, D**). For stage I & II tumors, the maximum positive count was 8, with a maximum total methylation value of 150. In contrast, stage III & IV tumors presented a maximum positive count of 14 and a maximum total methylation value of 600. These findings underscore the importance of our biomarker discovery process and validate the selection of a 14-CpG panel for the accurate identification of PAC patients.

**Figure 5:**
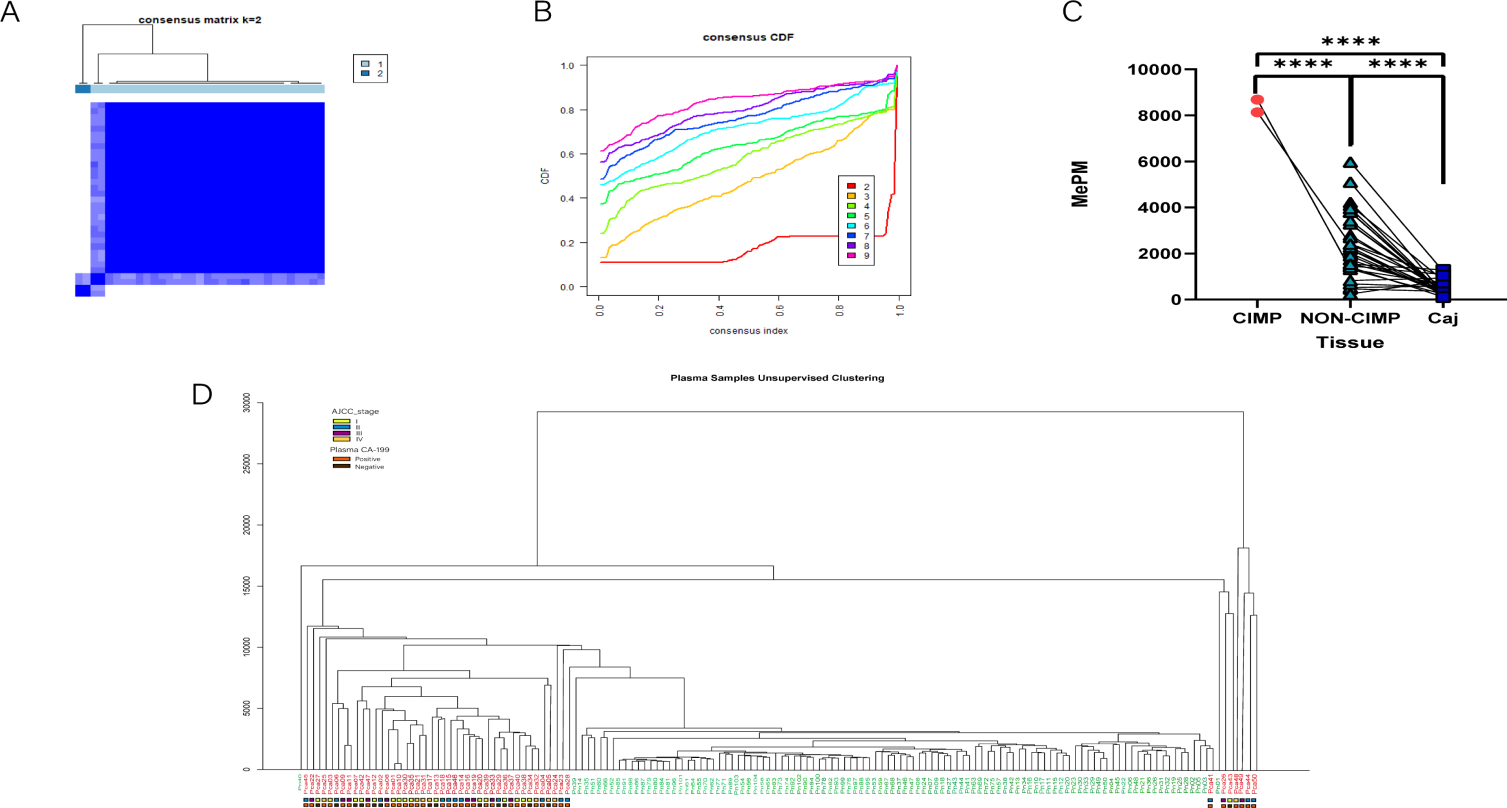
Tumor subtypes were identified via the methylation sequencing results of tissues and plasma. **(A)** The consensus matrix obtained when k = 2. The consistency values range from 0 to 1, where 0 means never clustering together (white), and one means always clustering together (dark blue). **(B)** The CDF curves for different values of k The value of k represents the number of clusters in the consensus cluster. When the optimal k value is reached, the area under the CDF curve does not significantly increase with increasing k value. **(C)** The total methylation values (MEPMs) of 120 mCGCGCGG-CpGs in different CIMP types of PAC and adjacent noncancerous tissues. ****P < 0.0001; two-tailed MWW test. **(D)** Unsupervised clustering results of PAC and healthy control plasma via MCTA-Seq.

The CDF and the relative change in the area under the CDF curve are presented in **Figures 5A, B**, respectively. In the consensus clustering analysis, determining the optimal k value is of utmost importance. Referring to the criteria established in existing literature, we defined the optimal k value as the point beyond which the area under the CDF curve shows minimal change with further increases in k. As a result, when k = 2, all tumor tissues were classified into two distinct subgroups: the CpG island methylation phenotype (CIMP) tumor cluster (n = 2) and the non-CIMP tumor cluster (n = 31) (**Figure 5C**). Although the CIMP and non-CIMP tumor clusters had similar median counts of positive CpG sites, the total methylation value of the 120 PAC biomarkers was significantly higher in the CIMP cluster (4809 for CIMP and 4692 for non-CIMP).

Among the 120 methylation markers for PAC, the range of positive marker counts was identical for patients in stages I and II (2-8), while it was 3-11 for stage III and 1-14 for stage IV. We can only conclude that a patient is in stage III or IV when the number of positive markers exceeds 8. Regarding the total positive methylation values of the 120 markers, the range was the same for patients in stages I and II (30 - 150), while it was 30 - 220 for stage III and 50 - 600 for stage IV. Similarly, we can only determine that a patient is in stage III or IV when the total positive methylation value exceeds 150. Correspondingly, we performed unsupervised hierarchical clustering on the plasma data. The results revealed that tumor patients and controls were almost completely separated into two distinct clusters (**Figure 5D**). This finding strongly indicates the powerful potential of MCTA - Seq for detection and classification in both tissue and blood samples, highlighting its significance in PAC research.

### 3.4 Specific biomarkers for each tumor

**Figure 6:**
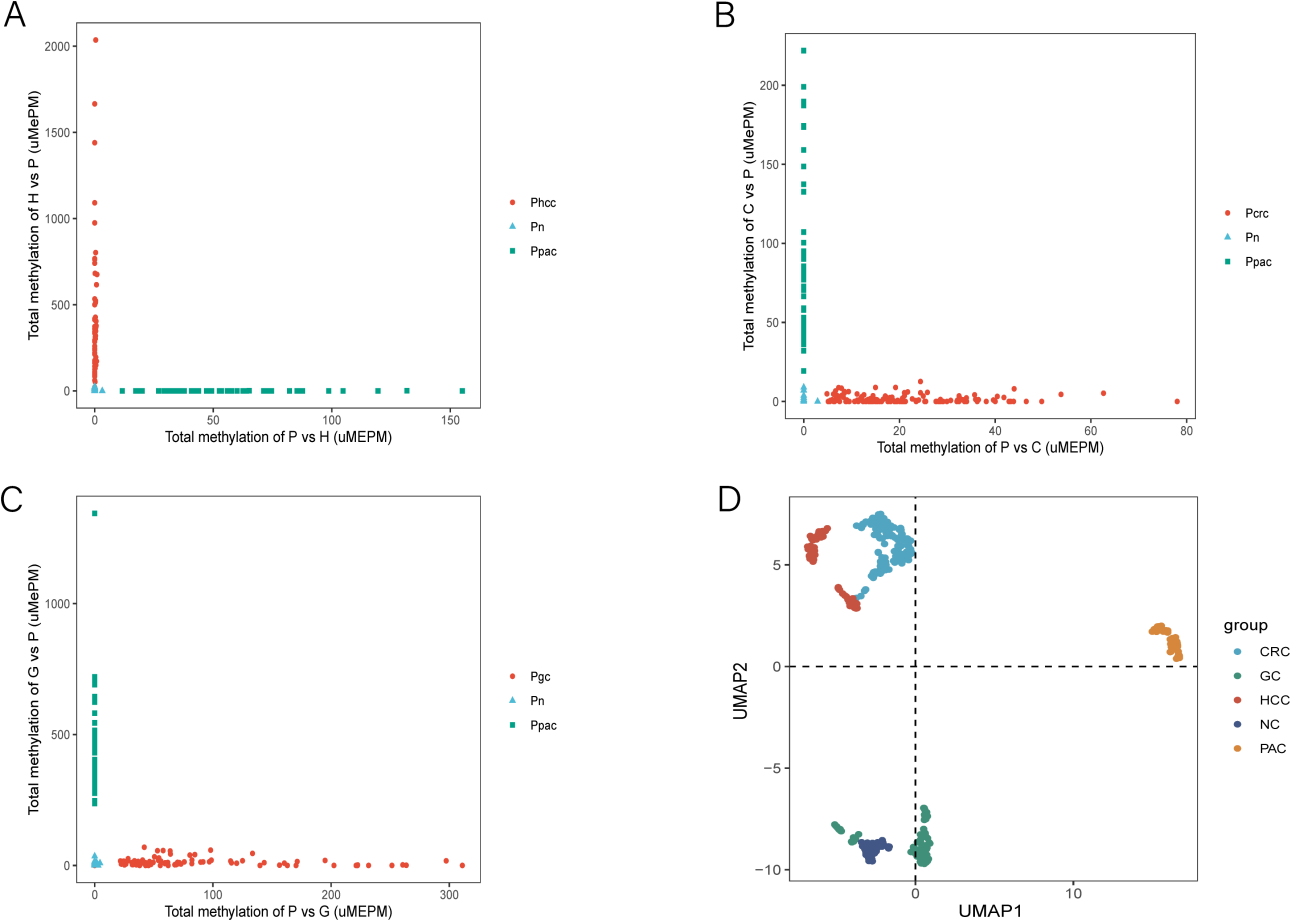
MCTA-Seq for discriminating PAC and HCC, PAC and CRC, and PAC and GC. **(A)** Total methylation values of the 621 PAC-specific mCGCGCGG-CpGs (P vs H) and the 36 HCC-specific mCGCGCGG-CpGs (H vs P) in the plasma of patients with PAC and HCC at different stages and control participants. **(B)** Total methylation values of the 148 PAC-specific mCGCGCGG-CpGs (P vs C) and the 10 CRC-specific mCGCGCGG-CpGs (C vs P) in the plasma of patients with PAC and CRC at different stages and control participants. **(C)** Total methylation values of the 1024 PAC-specific mCGCGCGG-CpGs (P vs G) and the 48 GC-specific mCGCGCGG-CpGs (G vs P) in the plasma of patients with PAC and GC in different stages and control participants. **(D)** Uniform manifold approximations and projections of PAC, HCC, CRC, GC and control participant plasma samples are indicated in the figure.

To determine whether MCTA-Seq could differentiate PAC from other cancer types in blood, we analyzed MCTA-Seq data from tissue and plasma samples of hepatocellular carcinoma (HCC; n=25 tissue, n=64 plasma), colorectal cancer (CRC; n=33 tissue, n=142 plasma), and gastric cancer (GC; n=28 tissue, n=89 plasma) patients, collected from previous studies (references 18-20). Similar to the findings in HCC, CRC, and GC, the 120 mCGCGCGG-CpGs associated with PAC were also predominantly positive in these other tumor types. This observation prompted us to search for more specific biomarkers to improve discrimination between cancer types. We performed differential methylation analysis to identify plasma biomarkers capable of distinguishing PAC from HCC, CRC, and GC. Through these comparisons, we identified 621 PAC-specific (P vs H) and 36 HCC-specific (H vs P) mCGCGCGG-CpGs;148 PAC-specific (P vs C) and 10 CRC-specific (C vs P) mCGCGCGG-CpGs; and1024 PAC-specific (P vs G) and 48 GC-specific (G vs P) mCGCGCGG-CpGs. For each set of biomarkers, we calculated the total methylation values for PAC and the respective tumor type, which clearly differentiated all four tumor types in blood (**Figures 6A-C**).

To further validate these findings, we employed Uniform Manifold Approximation and Projection (UMAP) analysis. This approach demonstrated clear separation between PAC and other tumor plasma samples, as well as between normal controls and patient plasma (**Figure 6D**). These analyses culminated in the identification of a panel of cfDNA methylation biomarkers capable of accurately distinguishing PAC from HCC, CRC, and GC.

### 3.5 Distinguishing the type of tumor in blood

Crucially, we next need to assess the possibility of MCTA-Seq simultaneously screening high-risk populations for HCC, CRC, GC and PAC, which is the core of our entire study.

**Figure 7:**
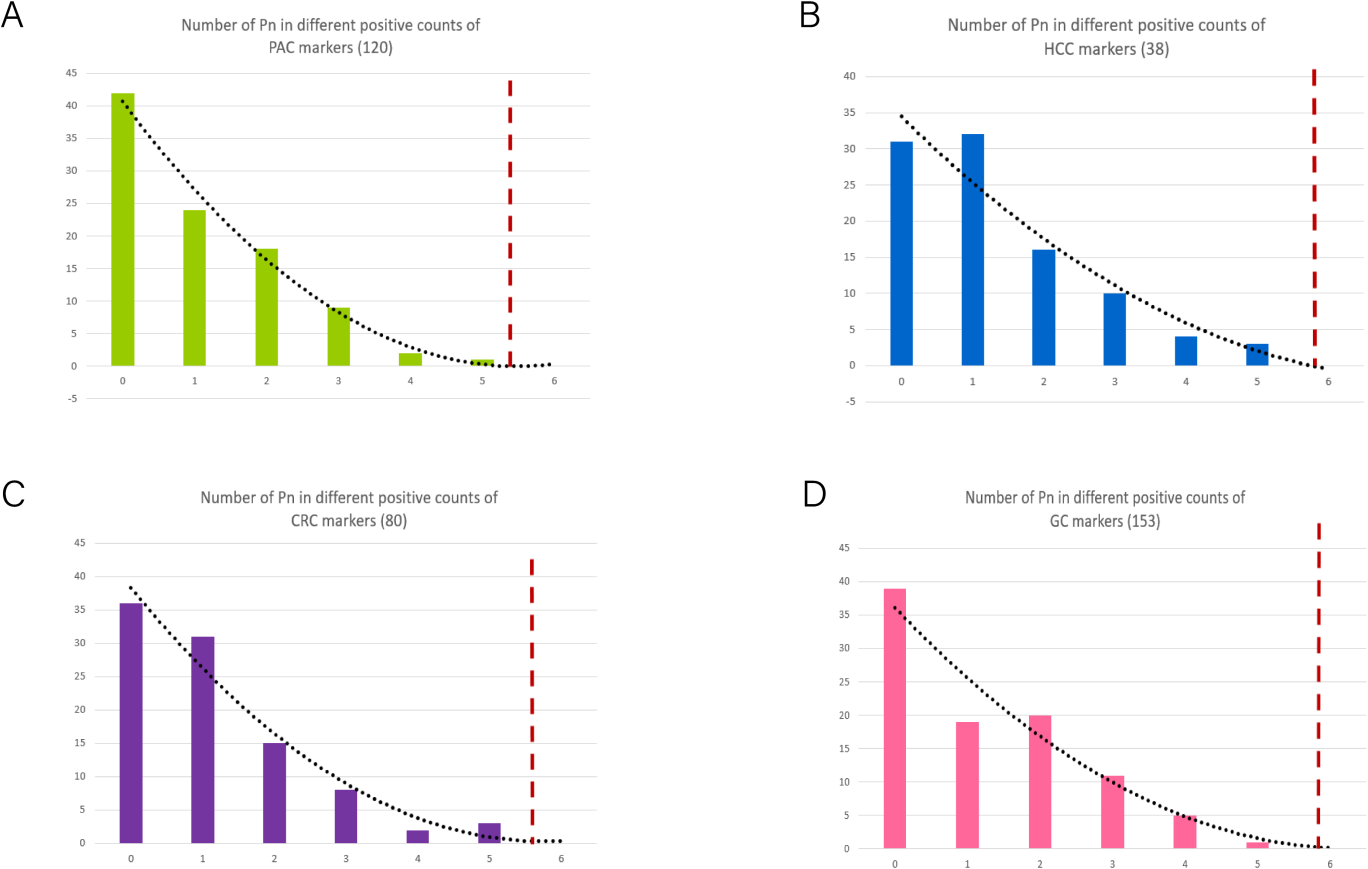
Determination of the cut-off values of positive biomarkers in different tumor types. **(A–D)** The process for establishing cut-off in PAC, HCC, CRC and GC patients according to the frequency of cancer diagnostic biomarkers in normal controls. The dashed lines indicate the cut-off in each set.

To achieve an optimal balance between sensitivity and specificity in screening, we established positive cut-off counts for diagnostic biomarkers specific to each tumor type. These cut-offs were determined through regression trend analysis using a preselection cohort of normal plasma samples (n=96). The resulting cut-off values were set at > 5 counts for HCC (n=38) (**Figure 7A**), CRC (n=80) (**Figure 7B**), GC (n=153) (**Figure 7C**), and PAC (n=120) (**Figure 7D**). In accordance of the established algorithm, we examined normal plasma and PAC in this study and HCC, CRC, and GC in previous studies. These findings highlight the robustness and reliability of the MCTA-Seq-based screening approach for simultaneously detecting multiple cancer types in high-risk populations. The high sensitivity and specificity achieved across all tumor types underscore the potential of this method for early and accurate cancer screening, which is critical for improving patient outcomes through timely intervention.

## 4 Discussion

Compared to other genomic DNA methylation detection methods, MCTA-Seq stands out as a semi-targeted approach, uniquely capable of directionally enriching hypermethylated CpG island genomic regions for high-throughput analysis. During the amplification process, MCTA-Seq enhances and concentrates signals from methylated CpG sites, significantly improving detection sensitivity and providing a more comprehensive and accurate depiction of methylation profiles, as evidenced in previous studies (5–8, 42–44). In this study, we applied MCTA-Seq to analyze tissue and peripheral blood samples from patients with PAC, enabling us to map the methylation landscape at the genomic level. Building on prior research, we achieved a significant milestone by successfully differentiating PAC from other cancer types, such as HCC, CRC, and GC.

MCTA-Seq targets the start sequence ‘CGCGCGGs’, the most prevalent among short tandem CpG types, including ‘CGGCGGCGGs’, ‘CGCGGCGGs’, and ‘CGCGGCGAs’. While this approach allows for the discovery of novel biomarkers on a genome-wide scale, it may miss sequences lacking ‘CGCGCGGs’. Our results identified several PAC methylation markers previously reported in blood, such as EVX2, IKZF1, and CCDN2, as well as novel biomarkers like DHX30, C3orf70, and BRF1. These overlapping markers across multiple studies underscore their diagnostic potential for PAC, while inconsistent markers may require further validation with larger sample sizes (45, 46). Previous experience suggests that combining cancer-specific biomarkers is more effective for distinguishing cancer origins than relying on tissue-specific markers, and MCTA-Seq excels in this regard (27, 28). We identified hundreds of differentially methylated biomarkers between PAC and other cancers (HCC, CRC, and GC). A classifier composed of these biomarkers achieved diagnostic accuracy exceeding 90%, which is particularly valuable for patients with cancer metastasis, as it aids in identifying the primary lesion.

MCTA-Seq demonstrates remarkable sensitivity and specificity, both surpassing 90%, for detecting stage I and II PAC. These results align with those reported by Shen SY et al., who used the limma-trend test statistic after large-scale sequencing to select the top 300 differentially methylated regions (DMRs) (47). In contrast, CA19-9, the only FDA-approved serum biomarker for PAC, has limited clinical utility due to its relatively low sensitivity (80%; 95% CI = 72-86%) and specificity (75%; 95% CI = 68-80%). Efforts to improve PAC diagnosis have explored combining CA19-9 with other biomarkers, such as Mucin 5AC (MUC5AC), though the effectiveness of such combinations remains under evaluation (48–50). Overall, the trend in PAC diagnosis is shifting toward the use of multi-biomarker panels rather than single biomarkers.

In recent years, bioinformatics methods have been widely used to identify diagnostic and prognostic markers (51–55). An increasing number of studies have investigated biomarkers related to cancer and inflammation identified through machine learning (56–59), single-cell analysis (60), and Mendelian randomization analysis (61–66). Beyond diagnosis, DNA methylation is closely linked to prognosis, cancer staging, and treatment response. Using consensus clustering analysis, we identified two distinct clusters with markedly different DNA methylation profiles, which significantly correlated with cancer stage and prognosis. While previous studies have explored methylation-based clustering in cancer patients, our research is the first to apply this approach specifically to PAC (67). Additionally, we developed a scoring system that clearly differentiates patients (scores > 0) from normal controls (scores < 0). These scoring and typing systems have accelerated clinical translation, and their integration into clinical practice is anticipated in the near future, offering significant benefits for patient care and management. Our findings clearly demonstrate that MCTA-Seq serves as a highly potent genome-wide DNA methylation detection method, particularly effective for the early noninvasive detection and discrimination of PAC. This outcome is highly consistent with our initial anticipations.

## 5 Conclusion

In this study, we identified 120 cfDNA methylation biomarkers in blood for the detection of PAC, which demonstrated excellent sensitivity and specificity. The innovative aspect of our study is the development of a methylation score and typing system specifically for PAC, which we believe holds great potential for clinical application. Furthermore, we have uncovered hundreds of differentially methylated cfDNA biomarkers that can effectively distinguish PAC from HCC, CRC, and GC with high specificity.

## Data Availability

The raw sequencing data were deposited in The Genome Sequence Archive for Human (GSA-Human) with the accession number HRA010563.

## Supplementary material

Supplementary Table 1 Baseline characteristics of study cohort.

Supplementary Table 2 120 Methylated cfDNA Biomarkers of Pancreatic cancer.

Supplementary Table 3 Specific biomarkers for PAC, HCC, CRC and GC.

Supplementary Table 4 Classification results from plasma samples in patients with PAC, non-PAC(HCC+CRC+GC) and normal control individuals.

## References

[1] Ibrahim J Peeters M Van Camp G Op de Beeck K. Methylation biomarkers for early cancer detection and diagnosis: Current and future perspectives. Eur J Cancer. (2023) 178:91–113. doi: 10.1016/j.ejca.2022.10.015 36427394

[2] Papanicolau-Sengos A Aldape K. DNA methylation profiling: an emerging paradigm for cancer diagnosis. Annu Rev Pathol. (2022) 17:295–321. doi: 10.1146/annurev-pathol-042220-022304 34736341

[3] Wang Y Wang J He J Ji B Pang Z Wang J. Comprehensive analysis of PRPF19 immune infiltrates, DNA methylation, senescence-associated secretory phenotype and ceRNA network in bladder cancer. Front Immunol. (2023) 14:1289198. doi: 10.3389/fimmu.2023.1289198 38022515 PMC10657824

[4] Wang Y Wang J Liu Y Wang X Ren M. Multidimensional pan-cancer analysis of HSPA5 and its validation in the prognostic value of bladder cancer. Heliyon. (2024) 10:e27184. doi: 10.1016/j.heliyon.2024.e27184 38496902 PMC10944199

[5] Ahn J Heo S Lee J Bang D. Introduction to single-cell DNA methylation profiling methods. Biomolecules. (2021) 11:1013. doi: 10.3390/biom11071013 34356635 PMC8301785

[6] Eleftheriou M Ruzov A. Modified forms of cytosine in eukaryotes: DNA (De)methylation and beyond. Methods Mol Biol. (2021) 2198:3–13. doi: 10.1007/978-1-0716-0876-0_1 32822018

[7] Foley JW Zhu SX West RB. Cost-effective DNA methylation profiling by FML-seq. Life Sci Alliance. (2023) 6:e202302326. doi: 10.1101/2023.01.13.523849 37775270 PMC10546043

[8] Li S Tollefsbol TO. DNA methylation methods: Global DNA methylation and methylomic analyses. Methods. (2021) 187:28–43. doi: 10.1016/j.ymeth.2020.10.002 33039572 PMC7914139

[9] Skvortsova K Stirzaker C Taberlay P. The DNA methylation landscape in cancer. Essays Biochem. (2019) 63:797–811. doi: 10.1042/EBC20190037 31845735 PMC6923322

[10] Tourancheau A Mead EA Zhang XS Fang G. Discovering multiple types of DNA methylation from bacteria and microbiome using nanopore sequencing. Nat Methods. (2021) 18:491–8. doi: 10.1038/s41592-021-01109-3 PMC8107137 33820988

[11] Yue X Xie Z Li M Wang K Li X Zhang X. Simultaneous profiling of histone modifications and DNA methylation via nanopore sequencing. Nat Commun. (2022) 13:7939. doi: 10.1038/s41467-022-35650-2 36566265 PMC9789962

[12] Stoffel EM Brand RE Goggins M. Pancreatic cancer: changing epidemiology and new approaches to risk assessment, early detection, and prevention. Gastroenterology. (2023) 164:752–65. doi: 10.1053/j.gastro.2023.02.012 PMC10243302 36804602

[13] Hou J Li X Xie KP. Coupled liquid biopsy and bioinformatics for pancreatic cancer early detection and precision prognostication. Mol Cancer. (2021) 20:34. doi: 10.1186/s12943-021-01309-7 33593396 PMC7888169

[14] Wood LD Canto MI Jaffee EM Simeone DM. Pancreatic cancer: pathogenesis, screening, diagnosis, and treatment. Gastroenterology. (2022) 163:386–402 e381. doi: 10.1053/j.gastro.2022.03.056 35398344 PMC9516440

[15] Luo G Jin K Deng S Cheng H Fan Z Gong Y. Roles of CA19-9 in pancreatic cancer: Biomarker, predictor and promoter. Biochim Biophys Acta Rev Cancer. (2021) 1875:188409. doi: 10.1016/j.bbcan.2020.188409 32827580

[16] Cao K Xia Y Yao J Han X Lambert L Zhang T. et al: Large-scale pancreatic cancer detection via non-contrast CT and deep learning. Nat Med. (2023) 29:3033–43. doi: 10.1038/s41591-023-02640-w PMC10719100 37985692

[17] Li C Liu T Liu Y Zhang J Zuo D. Prognostic value of tumour microenvironment-related genes by TCGA database in rectal cancer. J Cell Mol Med. (2021) 25:5811–22. doi: 10.1111/jcmm.v25.12 PMC8184694 33949771

[18] Wang Y Ma L He J Gu H Zhu H. Identification of cancer stem cell-related genes through single cells and machine learning for predicting prostate cancer prognosis and immunotherapy. Front Immunol. (2024) 15:1464698. doi: 10.3389/fimmu.2024.1464698 39267762 PMC11390519

[19] Zhu C Sun Z Wang J Meng X Ma Z Guo R. et al: Exploring oncogenes for renal clear cell carcinoma based on G protein-coupled receptor-associated genes. Discovery Oncol. (2023) 14:182. doi: 10.1007/s12672-023-00795-z PMC10564696 37816979

[20] Gao Y Zhao H An K Liu Z Hai L Li R. et al: Whole-genome bisulfite sequencing analysis of circulating tumour DNA for the detection and molecular classification of cancer. Clin Transl Med. (2022) 12:e1014. doi: 10.1002/ctm2.1014 35998020 PMC9398227

[21] Constancio V Nunes SP Moreira-Barbosa C Freitas R Oliveira J Pousa I. et al: Early detection of the major male cancer types in blood-based liquid biopsies using a DNA methylation panel. Clin Epigenet. (2019) 11:175. doi: 10.1186/s13148-019-0779-x PMC6889617 31791387

[22] Gao Q Lin YP Li BS Wang GQ Dong LQ Shen BY. et al: Unintrusive multi-cancer detection by circulating cell-free DNA methylation sequencing (THUNDER): development and independent validation studies. Ann Oncol. (2023) 34:486–95. doi: 10.1016/j.annonc.2023.02.010 36849097

[23] Wen L Li J Guo H Liu X Zheng S Zhang D. et al: Genome-scale detection of hypermethylated CpG islands in circulating cell-free DNA of hepatocellular carcinoma patients. Cell Res. (2015) 25:1376. doi: 10.1038/cr.2015.141 26620315 PMC4670997

[24] Zuo D Zhang J Liu T Li C Ning G. Claudin-1 is a valuable prognostic biomarker in colorectal cancer: A meta-analysis. Gastroenterol Res Pract. (2020) 2020:4258035. doi: 10.1155/2020/4258035 32855635 PMC7443231

[25] Sun Z Wang J Fan Z Yang Y Meng X Ma Z. et al: Investigating the prognostic role of lncRNAs associated with disulfidptosis-related genes in clear cell renal cell carcinoma. J Gene Med. (2024) 26:e3608. doi: 10.1002/jgm.3608 37897262

[26] Pang ZQ Wang JS Wang JF Wang YX Ji B Xu YD. et al: JAM3: A prognostic biomarker for bladder cancer via epithelial-mesenchymal transition regulation. Biomol BioMed. (2024) 24:897–911. doi: 10.17305/bb.2024.9979 38400838 PMC11293228

[27] Li J Zhou X Liu X Ren J Wang J Wang W. et al: detection of colorectal cancer in circulating cell-free DNA by methylated cpG tandem amplification and sequencing. Clin Chem. (2019) 65:916–26. doi: 10.1373/clinchem.2019.301804 31010820

[28] Ren J Lu P Zhou X Liao Y Liu X Li J. Genome-scale methylation analysis of circulating cell-free DNA in gastric cancer patients. Clin Chem. (2022) 68:354–64. doi: 10.1093/clinchem/hvab204 34791072

[29] Zuo D Li C Liu T Yue M Zhang J Ning G. Construction and validation of a metabolic risk model predicting prognosis of colon cancer. Sci Rep. (2021) 11:6837. doi: 10.1038/s41598-021-86286-z 33767290 PMC7994414

[30] Wang J Zuo Z Yu Z Chen Z Tran LJ Zhang J. Collaborating single-cell and bulk RNA sequencing for comprehensive characterization of the intratumor heterogeneity and prognostic model development for bladder cancer. Aging (Albany NY). (2023) 15:12104–19. doi: 10.18632/aging.205166 PMC10683618 37950728

[31] Li C Wirth U Schardey J Ehrlich-Treuenstätt VV Bazhin AV Werner J. An immune-related gene prognostic index for predicting prognosis in patients with colorectal cancer. Front Immunol. (2023) 14:1156488. doi: 10.3389/fimmu.2023.1156488 37483596 PMC10358773

[32] Zhang C Sun D Li C Liu Y Zhou Y Zhang J. Development of cancer-associated fibroblasts subtype and prognostic model in gastric cancer and the landscape of tumor microenvironment. Int J Biochem Cell Biol. (2022) 152:106309. doi: 10.1016/j.biocel.2022.106309 36174922

[33] Jiang S Yang X Lin Y Liu Y Tran LJ Zhang J. Unveiling Anoikis-related genes: A breakthrough in the prognosis of bladder cancer. J Gene Med. (2024) 26:e3651. doi: 10.1002/jgm.v26.1 38282152

[34] Liu T Li C Zhang J Hu H Li C. Unveiling efferocytosis-related signatures through the integration of single-cell analysis and machine learning: a predictive framework for prognosis and immunotherapy response in hepatocellular carcinoma. Front Immunol. (2023) 14:1237350. doi: 10.3389/fimmu.2023.1237350 37575252 PMC10414188

[35] Zhou W Yun Z Wang T Li C Zhang J. BTF3-mediated regulation of BMI1 promotes colorectal cancer through influencing epithelial-mesenchymal transition and stem cell-like traits. Int J Biol Macromol. (2021) 187:800–10. doi: 10.1016/j.ijbiomac.2021.07.106 34293363

[36] Jin W Yang Q Chi H Wei K Zhang P Zhao G. Ensemble deep learning enhanced with self-attention for predicting immunotherapeutic responses to cancers. Front Immunol. (2022) 13:1025330. doi: 10.3389/fimmu.2022.1025330 36532083 PMC9751999

[37] Wang Y He J Zhao Q Bo J Zhou Y Sun H. Evaluating the predictive value of angiogenesis-related genes for prognosis and immunotherapy response in prostate adenocarcinoma using machine learning and experimental approaches. Front Immunol. (2024) 15:1416914. doi: 10.3389/fimmu.2024.1416914 38817605 PMC11137278

[38] Zhai X Zhang H Xia Z Liu M Du G Jiang Z. et al: Oxytocin alleviates liver fibrosis via hepatic macrophages. JHEP Rep. (2024) 6:101032. doi: 10.1016/j.jhepr.2024.101032 38882603 PMC11177191

[39] Xiao J Lin H Liu B Xia Z Zhang J Jin J. Decreased S1P and SPHK2 are involved in pancreatic acinar cell injury. biomark Med. (2019) 13:627–37. doi: 10.2217/bmm-2018-0404 31157539

[40] Xiao J Huang K Lin H Xia Z Zhang J Li D. Mogroside II(E) inhibits digestive enzymes via suppression of interleukin 9/interleukin 9 receptor signalling in acute pancreatitis. Front Pharmacol. (2020) 11:859. doi: 10.3389/fphar.2020.00859 32587518 PMC7298197

[41] Zhang H Xia T Xia Z Zhou H Li Z Wang W. KIF18A inactivates hepatic stellate cells and alleviates liver fibrosis through the TTC3/Akt/mTOR pathway. Cell Mol Life Sci. (2024) 81:96. doi: 10.1007/s00018-024-05114-5 38372748 PMC10876760

[42] Fernandez-Delgado M Sirsat MS Cernadas E Alawadi S Barro S Febrero-Bande M. An extensive experimental survey of regression methods. Neural Netw. (2019) 111:11–34. doi: 10.1016/j.neunet.2018.12.010 30654138

[43] Gong T Borgard H Zhang Z Chen S Gao Z Deng Y. Analysis and performance assessment of the whole genome bisulfite sequencing data workflow: currently available tools and a practical guide to advance DNA methylation studies. Small Methods. (2022) 6:e2101251. doi: 10.1002/smtd.202101251 35064762 PMC8963483

[44] Paun O Verhoeven KJF Richards CL. Opportunities and limitations of reduced representation bisulfite sequencing in plant ecological epigenomics. New Phytol. (2019) 221:738–42. doi: 10.1111/nph.2019.221.issue-2 PMC6504643 30121954

[45] Vucic EA Wilson IM Campbell JM Lam WL. Methylation analysis by DNA immunoprecipitation (MeDIP). Methods Mol Biol. (2009) 556:141–53. doi: 10.1007/978-1-60327-192-9_10 19488876

[46] Garcia-Ortiz MV Cano-Ramirez P Toledano-Fonseca M Aranda E Rodriguez-Ariza A. Diagnosing and monitoring pancreatic cancer through cell-free DNA methylation: progress and prospects. biomark Res. (2023) 11:88. doi: 10.1186/s40364-023-00528-y 37798621 PMC10552233

[47] Wu H Guo S Liu X Li Y Su Z He Q. et al: Noninvasive detection of pancreatic ductal adenocarcinoma using the methylation signature of circulating tumour DNA. BMC Med. (2022) 20:458. doi: 10.1186/s12916-022-02647-z 36434648 PMC9701032

[48] Shen SY Singhania R Fehringer G Chakravarthy A Roehrl MHA Chadwick D. et al: Sensitive tumour detection and classification using plasma cell-free DNA methylomes. Nature. (2018) 563:579–83. doi: 10.1038/s41586-018-0703-0 30429608

[49] Yang J Xu R Wang C Qiu J Ren B You L. Early screening and diagnosis strategies of pancreatic cancer: a comprehensive review. Cancer Commun (Lond). (2021) 41:1257–74. doi: 10.1002/cac2.v41.12 PMC8696234 34331845

[50] Zhang J Wang Y Zhao T Li Y Tian L Zhao J. Evaluation of serum MUC5AC in combination with CA19-9 for the diagnosis of pancreatic cancer. World J Surg Oncol. (2020) 18:31. doi: 10.1186/s12957-020-1809-z 32028958 PMC7006398

[51] Deng Y Shi M Yi L Naveed Khan M Xia Z Li X. Eliminating a barrier: Aiming at VISTA, reversing MDSC-mediated T cell suppression in the tumor microenvironment. Heliyon. (2024) 10:e37060. doi: 10.1016/j.heliyon.2024.e37060 39286218 PMC11402941

[52] Zhang X Zhang P Cong A Feng Y Chi H Xia Z. Unraveling molecular networks in thymic epithelial tumors: deciphering the unique signatures. Front Immunol. (2023) 14:1264325. doi: 10.3389/fimmu.2023.1264325 37849766 PMC10577431

[53] Xia Z Chen S He M Li B Deng Y Yi L. Editorial: Targeting metabolism to activate T cells and enhance the efficacy of checkpoint blockade immunotherapy in solid tumors. Front Immunol. (2023) 14:1247178. doi: 10.3389/fimmu.2023.1247178 37575246 PMC10415066

[54] Zhang J Peng G Chi H Yang J Xie X Song G. CD8 + T-cell marker genes reveal different immune subtypes of oral lichen planus by integrating single-cell RNA-seq and bulk RNA-sequencing. BMC Oral Health. (2023) 23:464. doi: 10.1186/s12903-023-03138-0 37422617 PMC10329325

[55] Zhang X Zhuge J Liu J Xia Z Wang H Gao Q. et al: Prognostic signatures of sphingolipids: Understanding the immune landscape and predictive role in immunotherapy response and outcomes of hepatocellular carcinoma. Front Immunol. (2023) 14:1153423. doi: 10.3389/fimmu.2023.1153423 37006285 PMC10063861

[56] Zhang P Pei S Wu L Xia Z Wang Q Huang X. Integrating multiple machine learning methods to construct glutamine metabolism-related signatures in lung adenocarcinoma. Front Endocrinol (Lausanne). (2023) 14:1196372. doi: 10.3389/fendo.2023.1196372 37265698 PMC10229769

[57] Chi H Gao X Xia Z Yu W Yin X Pan Y. et al: FAM family gene prediction model reveals heterogeneity, stemness and immune microenvironment of UCEC. Front Mol Biosci. (2023) 10:1200335. doi: 10.3389/fmolb.2023.1200335 37275958 PMC10235772

[58] Ren Q Zhang P Lin H Feng Y Chi H Zhang X. A novel signature predicts prognosis and immunotherapy in lung adenocarcinoma based on cancer-associated fibroblasts. Front Immunol. (2023) 14:1201573. doi: 10.3389/fimmu.2023.1201573 37325647 PMC10264584

[59] Zhang S Jiang C Jiang L Chen H Huang J Gao X. et al: Construction of a diagnostic model for hepatitis B-related hepatocellular carcinoma using machine learning and artificial neural networks and revealing the correlation by immunoassay. Tumour Virus Res. (2023) 16:200271. doi: 10.1016/j.tvr.2023.200271 37774952 PMC10638043

[60] Liu J Zhang P Yang F Jiang K Sun S Xia Z. Integrating single-cell analysis and machine learning to create glycosylation-based gene signature for prognostic prediction of uveal melanoma. Front Endocrinol (Lausanne). (2023) 14:1163046. doi: 10.3389/fendo.2023.1163046 37033251 PMC10076776

[61] Hu Y Wang K Chen Y Jin Y Guo Q Tang H. Causal relationship between immune cell phenotypes and risk of biliary tract cancer: evidence from Mendelian randomization analysis. Front Immunol. (2024) 15:1430551. doi: 10.3389/fimmu.2024.1430551 39050844 PMC11266158

[62] Wang K Wang S Qin X Chen Y Chen Y Wang J. The causal relationship between gut microbiota and biliary tract cancer: comprehensive bidirectional Mendelian randomization analysis. Front Cell Infect Microbiol. (2024) 14:1308742. doi: 10.3389/fcimb.2024.1308742 38558852 PMC10978781

[63] Wang K Wang J Chen Y Long H Pan W Liu Y. Causal relationship between gut microbiota and risk of esophageal cancer: evidence from Mendelian randomization study. Aging (Albany NY). (2024) 16:3596–611. doi: 10.18632/aging.205547 PMC10929825 38364235

[64] Wang K Qin X Ran T Pan Y Hong Y Wang J. et al: Causal link between gut microbiota and four types of pancreatitis: a genetic association and bidirectional Mendelian randomization study. Front Microbiol. (2023) 14:1290202. doi: 10.3389/fmicb.2023.1290202 38075894 PMC10702359

[65] Liu R Wang K Guo X Wang Q Zhang X Peng K. A causal relationship between distinct immune features and acute or chronic pancreatitis: results from a mendelian randomization analysis. Pancreatology. (2024) 24:1219–28. doi: 10.1016/j.pan.2024.10.006 39419750

[66] Wang K Chen Y Li Y. Evaluating concordance and clinical utility of ctDNA profiling in advanced biliary tract cancer. J Hepatol. (2024) 24:2715–6. doi: 10.1016/j.jhep.2024.11.014 39551391

[67] Zhang E Shiori F Mu OY He J Ge Y Wu H. Establishment of novel DNA methylation-based prostate cancer subtypes and a risk-predicting eight-gene signature. Front Cell Dev Biol. (2021) 9:639615. doi: 10.3389/fcell.2021.639615 33708770 PMC7940376

